# Radiotherapy versus combination radiotherapy-bevacizumab for the treatment of recurrent high-grade glioma: a systematic review

**DOI:** 10.1007/s00701-021-04794-3

**Published:** 2021-04-02

**Authors:** Daniel P. Kulinich, John P. Sheppard, Thien Nguyen, Aditya M. Kondajji, Ansley Unterberger, Courtney Duong, Adam Enomoto, Kunal Patel, Isaac Yang

**Affiliations:** 1 Departments of Neurosurgery, University of California, Los Angeles, 300 Stein Plaza, Suite 562, Los Angeles, CA USA; 2 David Geffen School of Medicine, Los Angeles (UCLA), Los Angeles, CA USA; 3 Departments of Radiation Oncology, University of California, Los Angeles, Los Angeles, CA USA; 4 Departments of Head and Neck Surgery, University of California, Los Angeles, Los Angeles, CA USA; 5 Jonsson Comprehensive Cancer Center, Los Angeles, CA USA; 6 Los Angeles Biomedical Research Institute, Los Angeles, CA USA; 7 Harbor-UCLA Medical Center, West Carson, CA USA

**Keywords:** Radiotherapy, Radiosurgery, Recurrent, Glioma, Astrocytoma, Glioblastoma, Bevacizumab

## Abstract

**Background:**

High-grade gliomas (HGG) comprise the most common primary adult brain cancers and universally recur. Combination of re-irradiation therapy (reRT) and bevacizumab (BVZ) therapy for recurrent HGG is common, but its reported efficacy is mixed.

**Objective:**

To assess clinical outcomes after reRT ± BVZ in recurrent HGG patients receiving stereotactic radiosurgery (SRS), hypofractionated radiosurgery (HFSRT), or fully fractionated radiotherapy (FFRT).

**Methods:**

We performed a systematic review of PubMed, Web of Science, Scopus, Embase, and Cochrane databases, following the Preferred Reporting Items for Systematic Reviews and Meta-Analyses (PRISMA) guidelines. We identified studies reporting outcomes for patients with recurrent HGG treated via reRT ± BVZ. Cohorts were stratified by BVZ treatment status and re-irradiation modality (SRS, HFSRT, and FFRT). Outcome variables were overall survival (OS), progression-free survival (PFS), and radiation necrosis (RN).

**Results:**

Data on 1399 patients was analyzed, with 954 patients receiving reRT alone and 445 patients receiving reRT + BVZ. All patients initially underwent standard-of-care therapy for their primary HGG. In a multivariate analysis that adjusted for median patient age, WHO grade, RT dosing, reRT fractionation regimen, time between primary and re-irradiation, and re-irradiation target volume, BVZ therapy was associated with significantly improved OS (2.51, 95% CI [0.11, 4.92] months, *P* = .041) but no significant improvement in PFS (1.40, 95% CI [− 0.36, 3.18] months, *P* = .099). Patients receiving BVZ also had significantly lower rates of RN (2.2% vs 6.5%, *P* < .001).

**Conclusions:**

Combination of reRT + BVZ may improve OS and reduce RN rates in recurrent HGG, but further controlled studies are needed to confirm these effects.

**Supplementary Information:**

The online version contains supplementary material available at 10.1007/s00701-021-04794-3.

## Introduction

Glioblastoma (GBM) and anaplastic astrocytoma comprise the majority of gliomas. While incurable, a growing armamentarium of treatment options requires complex interdisciplinary decision-making to determine optimal management strategies based on individual patient-care goals. Classically, the World Health Organization (WHO) has histologically classified high-grade gliomas (HGGs) as WHO grade III gliomas, which include anaplastic astrocytomas (AA) and oligodendrogliomas (AOA), and WHO grade IV glioblastoma [41]. GBM accounts for approximately 60% of HGG and 50% of all malignant brain tumors and portends the poorest prognosis [19]. Since then, WHO glioma classification has been re-examined and re-defined according to various genetic markers, mainly IDH mutation and 1p/19q deletion status [41]. The current standard-of-care for GBM involves a multimodal approach, including surgical resection of the primary lesion, chemotherapy with temozolomide, and adjuvant radiotherapy (typically involving conventional external beam radiation therapy, EBRT). Landmark studies by Stupp and colleagues demonstrated improved 2-year survival to 27.2% from 10.9% when adding EBRT to temozolomide (TMZ) chemotherapy in primary GBM [71, 72]. Even with modern treatment regimens, however, HGG recurrence is virtually inevitable. Estimated post-recurrence survival in AA and GBM patients is approximately 10 and 6 months, respectively [72]. Thus, there is a need for further clinical investigation of survival outcomes to optimize treatment protocols for recurrent HGG.

Eighty percent of recurrent HGG tumors appear within 2 cm of the initial contrast-enhanced primary lesion [48]. Recent advances in imaging and radiotherapy techniques allow irradiation with higher doses, improved local tumor control, and sparing of adjacent tissue [1]. Several fractionation regimens are available, which offer flexibility between the number of treatment sessions and maximum radiation doses required, including stereotactic radiosurgery (SRS), hypofractionated stereotactic radiotherapy (HFSRT), and fully fractionated radiotherapy (FFRT) [64, 66]. HFSRT and FFRT regimens effectively target larger-volume lesions with lower doses administered over cumulative fractions, while SRS may offer a favorable radiation modality for smaller lesions [56]. Recurrent HGG is regularly treated with a combination of repeat surgical resection, systemic cancer therapies, and radiosurgery. Re-resection and repeat radiation therapy (reRT) improve overall survival (OS) and progression-free survival (PFS) in recurrent HGG; however, lesion size and proximity to eloquent tissue may limit reRT use [64]. Adjuvant chemotherapy and radio-sensitizing agents may further improve outcomes in the setting of reRT, but their effect on survival remains inconclusive [17, 62]. Furthermore, the reported efficacy of traditional chemotherapy agents such as TMZ is limited to patients with non-resistant, MGMT-methylated primary, or recurrent tumors [53, 65]. Overall, despite a plethora of new therapeutic alternatives, there remains no established standardized treatment protocol for recurrent HGG [35, 64].

One promising avenue for glioma treatment involves immunotherapy targeting tumor blood supply. HGG survives, differentiates, and grows well in hypoxic niches, which upregulates a conglomerate of molecular factors in the glial tumor cells, including hypoxic inhibitory factor (HIF)-1 and 2 [2]. HIF is a potent inducer of vascular endothelial growth factor (VEGF), a transcription factor key to promoting vasculogenesis. Neurovasculature in hypoxic tumor niches is tortuous and aberrant; it jeopardizes the blood-brain barrier and promotes further damage, edema, and necrosis. Anti-VEGF therapy with bevacizumab (BVZ) can reduce aberrant vasculogenesis, reduce radiation necrosis, and improve outcomes in clinical studies of HGG [5, 27, 28, 40]. Beyond its use in primary lesions, BVZ is combined with reRT (reRT + BVZ) to treat recurrent HGG. Recent studies have found that reRT + BVZ regimens are well tolerated, reduce radiation necrosis (RN) [23], and improve survival outcomes in HGG patients [8, 18, 29]. However, due to mixed results [14, 23], there remains no consensus on the utility of reRT + BVZ regimens for HGG. Here, we present a systematic review that synthesizes data from the published literature to assess the efficacy of reRT + BVZ treatment for recurrent HGG compared to reRT alone.

## Methods

Adherence to the Preferred Reporting Items for Systematic Reviews and Meta-Analyses (PRISMA) (www.prisma-statement.org) was maintained throughout this study. We indexed peer-reviewed abstracts and articles published between 1990 and 2019 in the following databases: PubMed, Scopus, Embase, Cochrane, and Web of Science. The last electronic search was completed in November 2019. An intersectional Boolean-search was performed to screen for articles with search terms including “high-grade glioma,” “glioblastoma,” “recurrent,” or “irradiation” AND (or NOT) “bevacizumab.” For inclusion in our systematic review, we required that articles met the following eligibility criteria: full-text, English-language clinical trials, prospective, or retrospective studies of patients with histologically proven, recurrent HGG who had initially received standard surgery and chemoradiation therapy for their primary lesion, with eventual tumor recurrence treated with either reRT or reRT + BVZ. Articles describing patients who received additional systemic therapies with reRT other than standard chemotherapy or BVZ, or who received reRT modalities other than SRS, HFSRT, or FFRT (e.g., brachytherapy), were excluded.

We further queried the bibliographies of identified manuscripts to screen for additional articles appropriate for review that could have escaped our electronic search. Some publications reported multiple treatment cohorts (e.g., both reRT and reRT + BVZ); in these cases, we separately extracted corresponding data for each treatment regimen. Where applicable, we required that authors controlled for demographic variables between groups in studies reporting aggregate demographic data across multiple treatment groups. Studies without reported demographic comparisons were excluded. Some articles reported overall survival data for WHO grades III/IV individually without reporting cumulative outcome data; in these cases, we only included data for GBM patients.

The following measures were extracted from included studies: (1) patient demographics (age, sex, and Karnofsky Performance Scale (KPS) at start of reRT), (2) WHO grade (III/IV), (3) primary RT and other adjuvant therapy parameters, (4) reRT and details of other therapies administered after recurrence, (5) latency from initial RT to reRT, (6) planned tumor volume for reRT (PTV). Extracted outcome measures included (1) overall survival after reRT (OS), (2) progression-free survival after reRT (PFS), (3) rates of radiation necrosis (RN), and (4) treatment-induced toxicity rates. For studies with multiple reRT protocols that qualified under a specific modality (SRS if < 5 fractions administered, HFSRT if 5–10 fractions administered, or FFRT if > 10 fractions administered), radiation dosage was computed as the average of the individual regimens. For comparative purposes, we converted radiation doses to reflect an equivalent total dose in 2 Gy fractions (EQD2) utilizing a linear-quadratic model with *α*/*β* = 2 [24]. Of note, IDH mutation and 1p/19q deletion status were not extracted since most publications did not classify according to the updated WHO glioma classification [41].

### Statistical analysis

We utilized unpaired Welch *t*-tests and Fischer exact tests for pairwise comparisons of continuous and binary variables. BVZ treatment and reRT fractionation modality were the two primary explanatory variables-of-interest. To compare OS and PFS between BVZ treatment groups (reRT vs. reRT + BVZ) and between reRT radiation modalities (SRS vs. HFSRT vs. FFRT), we used weighted Welch *t*-tests in order to weigh individual studies appropriately by their relative sample size when estimating pooled differences. Finally, we employed multivariate linear regression analysis to evaluate the amount of variance in OS and PFS explained collectively by all explanatory variables considered (median age at reRT, GBM diagnosis, BVZ treatment status, reRT fractionation modality, time between initial RT and reRT, planned target volume (PTV) for reRT, and EQD2 at both initial- and re-irradiation). All statistical analysis was performed in R, using the R core utilities in addition to the *weights* package for R [54, 59]. Statistical significance is defined as *P* <.05.

To further assess the quality of reviewed studies and the validity of our systematic review, we used standard meta-analytic methods to quantify cross-study heterogeneity and assess the risk of study bias (RoB) on OS and RN outcomes. We separately fit generalized linear mixed models within each treatment group that incorporated random study effects. These models allowed for the calculation of *H*, *τ*^2^, and *I*^2^, standard, and closely related metrics for quantifying heterogeneity in a meta-analysis. Statistical significance of study heterogeneity was determined for each treatment group based upon Cochran’s *Q* statistic. To assess the potential influence of RoB, we determined a numeric RoB score for each reviewed study which incorporated key factors that could increase each study’s susceptibility to bias. Estimation of RoB scores is fully described in Supplementary Table 3 and considered the overall rigor of described study methodology, whether studies were prospective, randomized, or blinded, as well as the variability in patients’ tumor subtypes, chemotherapy, reRT, immunotherapy regimens, stringency of RN diagnosis, and adequacy of follow-up. Regression analyses were performed for each treatment group to detect any associations between RoB score and reported outcomes. We used simple linear regression (i.e., Pearson’s correlations) to assess the impact of RoB on OS or PFS within each treatment group, given the limited number of studies reporting standard errors or confidence intervals for medial survival estimates. For RN outcomes, we used meta-regression to assess the impact of RoB score as this more robust approach also accounted for random study effects. Meta-analyses were performed using the *meta*, *metafor*, and *metamedian* packages for R [45, 63, 75]. The *metamedian* package was used to quantify heterogeneity in reported OS outcomes for studies which at minimum reported the range (min and max) of survival times in addition to medial survival, using quantile estimation methods described by McGrath et al. [44, 45]. We did not attempt to quantify heterogeneity in reported PFS outcomes, given the paucity of studies amenable to quantile estimation.

## Results

**Fig. 1:**
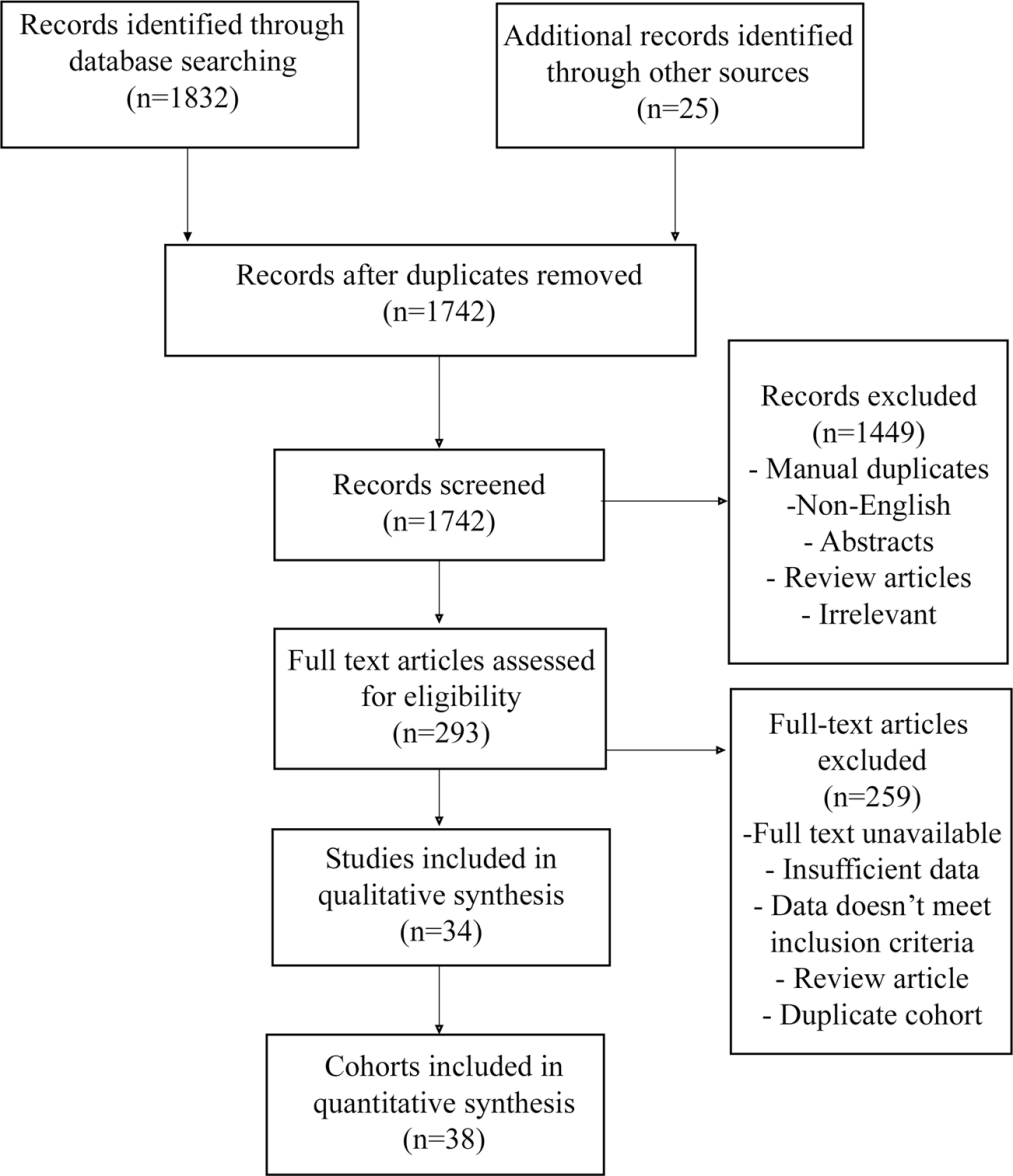
PRISMA flow diagram

Our combined electronic and manual bibliographic search identified 1742 articles in total before eligibility screening. Of these, 293 articles survived to the full-text review stage, of which 34 papers (2%) were deemed eligible for inclusion in our review (Fig. 1). Among the included studies, 26 papers reported data on patients receiving reRT alone, and 12 papers reported data on patients receiving reRT + BVZ **(**Tables 1 and 2) [3, 6–8, 10, 13–16, 18, 20, 22, 23, 26, 29–31, 33, 34, 36, 37, 42, 46, 51, 57, 61, 67–69, 74, 76, 77, 79, 80].

**Table 1:** Studies on re-irradiation with bevacizumab for recurrent high-grade glioma

Study	Modality	Patients (grade III/IV)	Median age (y)	Initial radiation* (Gy)	FU (mo)	Re-irradiation		Median KPS	Median PTV (cm^3^)	Median OS (mo)	Median PFS (mo)	RN (*N*)	Total toxicity (*N*)
						*(Gy)	EQD2 (Gy)						
Fleischmann 2019 [23]	FFRT	124 (29/95)	51	60/2	17	36/2	36	80	117	9.0	5.0	6	12
Palmer^γ^ 2018 [51]	FFRT	68 (14/48)	57	60/2	11	35/3.5	48	80	35	13.9	-	0	7
Palmer^γ^ 2018 [51]	FFRT	50 (5/39)	54	60/2	8	35/3.5	48	80	35	13.3	-	0	7
Schernberg 2017 [61]	FFRT	35 (11/24)	57	60/2	22	56/3.2	83	-	104	10.5	6.7	0	0
Back 2015 [3]	FFRT	18 (5/13)	50	60/2	15	35/2.0	30	-	136	10.0	-	1	2
Hundsberger 2013 [34]	FFRT	10 (4/6)	45	60/2	41	42/2.6	58	70	190	8.4	5.7	0	4
Yasuda 2018 [79]	HFSRT	29 (7/22)	46	60/2	19	42/6	84	80	34	10.4	5.6	0	3
Minniti 2015 [46]	HFSRT	26 (7/19)	50	60/2	13	25/5	44	-	31	11.0	-	0	4
Gutin 2009 [29]	HFSRT	25 (5/20)	56	59/2	15	30/6	60	80	34	12.5	7.3	0	31
Clarke 2017 [14]	SRS	15 (5/10)	63	60/2	-	33/11	107	90	-	13.0	7.0	1	14
Cabrera 2013 [8]	SRS	15 (7/8)	53	60/2	20	15/15	56	90	3	14.4	3.9	0	15
Cuneo 2012 [18]	SRS	42 (9/33)	47	60/2	21	17/9	48	80	5	11.2	5.2	2	17

*FFRT*, fully fractionated radiotherapy; *HFSRT*, hypofractionated stereotactic radiotherapy; *SRS*, stereotactic radiosurgery; *FU*, median latency between initial and re-irradiation; *EQD2*, equivalent dose in 2Gy per fraction; *KPS*, Karnofsky performance scale; *PTV*, planned tumor volume; *OS*, overall survival from re-irradiation; *PFS*, progression-free survival from re-irradiation; *RN*, radiation necrosis; *y*, year; *mo*, months; *N*, number

*Total dose/fraction dose

^γ^Same publication with separate cohorts

**Table 2:** Studies on re-irradiation alone for recurrent high-grade glioma

Study	Modality	Patients (grade III/IV)	Median age (y)	Initial radiation* (Gy)	FU (mo)	Re-irradiation		Median KPS	Median PTV (cm^3^)	Median OS (mo)	Median PFS (mo)	RN (*N*)	Total toxicity (*N*)
						*(Gy)	EQD2 (Gy)						
Fleischmann 2019 [23]	FFRT	37 (8/29)	51	60/2	18	36/2	36	80	122	9.0	5.0	5	14
Hundsberger 2013 [34]	FFRT	4 (2/2)	45	60/2	41	42/2.6	58	70	190	14.3	3.7	1	-
Combs 2005 [17]	FFRT	53 (0/53)	55	57/2	10	36/2	36	-	49	8.0	5.0	0	-
Gigliotti 2018 [26]	HFSRT	25 (5/25)	54	60/2	18	25/5	44	-	10	9.0	-	0	-
Zemlin 2018 [80]	HFSRT	41 (6/35)	56	60/2	22	31/4	30	-	63	6.7	4.3	2	-
Holt 2016 [31]	HFSRT	34 (0/34)	60	60/2	14	23/3	30	-	9	10.9	7.1	1	5
Dincoglan 2015 [20]	HFSRT	28 (0/28)	56	60/2	11	25/5	44	80	37	10.3	5.8	3	8
Ciammella 2013 [13]	HFSRT	15 (0/15)	52	60/2	11	25/5	44	90	-	9.5	-	0	-
Vordermark 2005 [76]	HFSRT	14 (0/14)	50	60/2	19	30/5	53	90	15	7.9	4.9	0	19
Voynov 2002 [77]	HFSRT	10 (4/4)	48	60/2	18	30/5	53	80	-	10.1	-	6	-
Hudes 1999 [33]	HFSRT	20 (1/19)	52	60/2	3	30/3	38	80	-	10.5	-	0	2
Shepherd 1997 [67]	HFSRT	21 (11/10)	37	55/2	29	35/3	53	-	-	10.7	-	4	0
Bir 2015 [6]	SRS	36 (0/36)	53	60/2	7	28/28	210	-	-	7.3	-	2	14
Pinzi 2015 [57]	SRS	128 (40/88)	51	60/2	15	20/10	69	-	5	11.5	-	7	19
Martinez-Carrillo 2014 [42]	SRS	87 (41/46)	49	60/2	14	18/18	90	83	-	10.0	-	0	-
Khalil 2013 [36]	SRS	50 (16/34)	58	60/2	10	15/15	64	70	-	11.4	8.6	7	10
Cuneo 2012 [18]	SRS	21 (5/16)	48	60/2	19	17/9	47	80	6	3.9	2.1	4	11
Skeie 2012 [69]	SRS	32 (0/32)	51	60/2	20	31/31	256	73	-	12.0	-	0	2
Elliott 2011 [22]	SRS	26 (10/16)	60	60/2	8	30/30	240	90	-	13.5	-	2	3
Torok 2011 [74]	SRS	14 (0/14)	58	60/2	13	24/12	84	-	-	10.0	5.0	0	0
Biswas 2009 [7]	SRS	33 (0/33)	58	60/2	9	15/15	64	-	-	6.7	4.3	1	-
Kong 2008 [37]	SRS	65 (0/65)	49	60/2	-	16/16	72	70	11	13.0	4.6	22	-
Combs 2005 [15]	SRS	32 (0/32)	56	54/2	10	15/15	64	-	10	10.0	5.0	0	0
Hall 1995 [30]	SRS	35 (8/26)	48	60/2	8	20/20	110	70	28	8.0	-	5	0
Shrieve 1995 [68]	SRS	76 (4/72)	46	60/2	10	13/13	49	80	-	10.2	-	0	-
Chamberlain 1994 [10]	SRS	20 (14/6)	34	60/2	11	14/14	52	80	-	8.0	4.0	0	7

*FFRT*, fully fractionated radiotherapy; *HFSRT*, hypofractionated stereotactic radiotherapy; *SRS*, stereotactic radiosurgery; *FU*, median latency between initial and re-irradiation; *EQD2*, equivalent dose in 2Gy per fraction; *KPS*, Karnofsky performance scale; *PTV*, planned tumor volume; *OS*, overall survival from re-irradiation; *PFS*, progression-free survival from re-irradiation; *RN*, radiation necrosis; *y*, year; *mo*, months; *N*, number

*Total dose/fraction dose

### Demographic variables

#### reRT group

We identified 954 patients receiving reRT alone. Ninety-four patients (10%) received FFRT, 206 (21%) received HFSRT, and 654 (69%) received SRS. Among 862 patients for whom gender was reported, 492 were male and 370 were female (*M* = 57%, *F* = 43%). Specifically, FFRT had 39 female patients (43%), HFSRT had 74 female patients (42%), and SRS had 257 female patients (43%). Among patients receiving reRT, 779 (82%) patients had GBM, and 175 (18%) had WHO grade III gliomas. reRT patients had a median Karnofsky Performance Score (KPS) of 80 (range: 40–100) (Table 3).

**Table 3:** Treatment group demographics

Modality	Patients		Age			WHO glioma grade			Karnofsky Performance Score		
	*N* (M/F)	*P* value*	Mean	SE	*P* value*	III	IV	*P* value* [95% CI]	Median	Range	*P* value*
Cumulative^i^	954 (492/370)	-	51.3	6.3	-	175 (18.3%)	779 (81.7%)	-	80	40–100	-
FFRT^i^	94 (50/39)	-	50.3	5.0	-	10 (10.6%)	84 (89.4%)	-	75	60–100	-
HFSRT^i^	206 (99/74)	-	51.6	6.5	-	27 (13.1%)	179 (86.9%)	-	80	60–100	-
SRS^i^	654 (343/257)	-	51.3	6.8	-	138 (21.1%)	516 (78.9%)	-	80	40–100	-
Cumulative^ii^	445 (299/154)	.002	52.4	5.3	.60	108 (24.3%)	337 (75.7%)	.012 [0.53, 0.93]	80	40–100	.48
FFRT^ii^	293 (195/106)	.17	52.3	4.7	.57	68 (23.2%)	225 (76.8%)	.0076 [0.17, 0.82]	80	40–100	.22
HFSRT^ii^	80 (42/24)	.38	50.7	5.0	.83	19 (23.8%)	61 (76.3%)	.032 [0.24, 0.993]	80	70–100	.21
SRS^ii^	72 (55/17)	.002	54.3	8.1	.51	21 (29.2%)	51 (70.8%)	.13 [0.37, 1.18]	90	50–100	.042

*M*, male; *F*, female; *SE*, standard error; *WHO*, World Health Organization; *FFRT*, fully fractionated radiotherapy; *HFSRT*, hypofractionated stereotactic radiotherapy, *SRS*; stereotactic radiosurgery; *CI*, confidence interval; “Cumulative” denotes combined statistic for all fractionation groups within a treatment group; *mo*, months; *N*, number

*Pairwise comparison between treatment groups

^i^Re-irradiation only group

^ii^Re-irradiation with bevacizumab group

#### reRT + BVZ group

Four hundred forty-five patients receiving reRT + BVZ were identified; 293 (66%) received FFRT, 80 (18%) received HFSRT, and 72 (16%) received SRS. Among 453 patients for whom gender was reported, 299 were male and 154 were female (*M* = 66%, *F* = 34%). Specifically, FFRT had 106 female patients (35%, *P* = .17), HFSRT had 24 female patients (36%, *P* = .38), and SRS had 17 female patients (24%, *P* = .002). Among patients receiving reRT + BVZ, 337 (76%) had GBM, and 108 (24%) had WHO grade III gliomas, with a median KPS of 80 (range: 40–100) **(**Table 3).

### Treatment parameters

All patients had either WHO grade III or IV tumors and had previously received primary surgical resection followed by RT with a median cumulative dose of 60 Gy and a median fractional dose of 2 Gy. All patients received chemotherapy for their primary lesion, with the most received agent being TMZ (> 54%). Patients receiving reRT alone underwent salvage reRT after a mean latency of 13.4 ± 5.3 months (range: 3–40 months), compared to 16.2 ± 5.9 months (range: 8–40 months, *P* = .18) for patients receiving reRT + BVZ (Table 4). reRT + BVZ patients received adjuvant BVZ dosed at 10 mg/kg once every two weeks for a range of 2–12 cycles.

**Table 4:** Treatment parameters

Modality	Re-irradiation total dose (Gy)				Total dose (Gy)			FU (mo)			Planned tumor volume (cm^3^)			
	Mean (EQD2)	SE (EQD2)	*P* value*	*P* value**	Mean	SE	*P* value*	Mean	SE	*P* value*	Mean	SE	*P* value*	*P* value**
Cumulative^i^	24.7 (76.5)	8.0 (56.9)	-	-	84.2	6.3	-	13.4	5.3	-	42.6	55.2	-	-
FFRT^i^	37.9 (36.9)	3.2 (5.4)	-	FFRT vs. HFSRT *P* = .43	96.9	5.0	-	14.5	7.3	-	120.5	70.5	-	FFRT vs. HFSRT *P* = .15
HFSRT^i^	28.2 (40.1)	3.8 (9.2)	-	HFSRT vs. SRS *P* = .0053	87.7	6.5	-	16.4	7.6	-	26.6	23.0	-	HFSRT vs. SRS *P* = .11
SRS^i^	19.7 (93.8)	6.2 (62.0)	-	SRS vs. FFRT *P* = .0058	79.3	6.8	-	12.2	3.1	-	11.9	9.3	-	SRS vs. FFRT *P* = .089
Cumulative^ii^	33.1 (51.3)	11.1 (11.0)	-	-	93.0	5.3	.60	16.2	5.9	.18	65.8	61.1	.34	-
FFRT^ii^	39.7 (46.5)	8.2 (16.3)	.20	FFRT vs. HFSRT *P* = .22	99.7	4.7	.57	15.4	5.6	.85	102.9	60.2	.91	FFRT vs. HFSRT *P* = .027
HFSRT^ii^	32.3 (64.4)	8.7 (20.6)	.13	HFSRT vs. SRS *P* = .29	92.1	8.9	.34	15.6	2.9	.35	33.0	1.7	.83	HFSRT vs. SRS *P* < .001
SRS^ii^	20.6 (61.9)	8.2 (22.8)	.088	SRS vs. FFRT *P* = .61	80.6	8.2	.78	20.5	0.6	< .001	3.8	1.0	.14	SRS vs. FFRT *P* = .0053

*EQD2*, equivalent dose in 2Gy per fraction; *SE*, standard error; *FFRT*, fully fractionated radiotherapy; *HFSRT*, hypofractionated stereotactic radiotherapy; *SRS*, stereotactic radiosurgery; “total dose,” sum of initial and irradiation doses not adjusted to EQD2; *FU*, latency between irradiations; *mo*, months; “cumulative” denotes combined statistic for all fractionation groups within a treatment group

*Pairwise comparison between treatment groups

**Pairwise comparison within a treatment group

^i^Re-irradiation only group

^ii^Re-irradiation with bevacizumab group

Within the reRT group, patients receiving SRS incurred lower cumulative doses at reRT (19.7 ± 6.2 Gy) than patients receiving HFSRT (28.2 ± 3.8 Gy, *P* = .0053) or FFRT (37.9 ± 3.2 Gy; *P* = .0058). There were no differences in total dose at re-irradiation among patients receiving different modalities within the reRT + BVZ treatment group (Table 4). PTV was comparable between reRT and reRT + BVZ groups after stratifying by reRT modality. PTV for reRT vs. reRT + BVZ patients averaged 120.5 cm^3^ vs. 102.9 cm^3^ for FFRT (*P* = .91), 26.6 cm^3^ vs. 33.0 cm^3^ for HFSRT (*P* = .83), and 11.9 cm^3^ vs. 3.8 cm^3^ for SRS (*P* = .14), respectively (Table 4). However, within the reRT + BVZ group, PTV was significantly different between each radiation modality group (Table 4). The latency between initial radiotherapy and re-irradiation only differed between reRT and reRT + BVZ patients who received SRS, which averaged 20.5 months for reRT + BVZ patients compared to 12.2 months for reRT patients (*P* < .001). Other demographic variables and radiation parameters did not differ significantly by treatment group or reRT modality (Tables 3 and 4).

### Clinical outcomes

The reRT treatment group had a mean OS of 9.9 ± 2.1 months, with PFS of 5.2 ± 1.6 months and RN rate of 9.5% (95% CI [7.7%, 11.6%]). In comparison, the reRT + BVZ group had a mean OS of 11.2 ± 2.1 months (*P* = .057), with PFS of 5.6 ± 1.0 months (*P* = .55), and an average RN rate of 2.2% (95% CI [1.1%, 4.0%], *P* < .001) (Table 5).

**Table 5:** Clinical outcomes

Modality	OS (mo)				PFS (mo)				RN (%)			
	Mean	SE	*P* value*	*P* value**	Mean	SE	*P* value*	*P* value**	Mean	95% CI	*P* value*	*P* value**
Cumulative^i^	9.9	2.1	-	-	5.2	1.6	-	-	6.5	[3.1, 9.9]	-	-
FFRT^i^	8.7	1.6	-	FFRT vs. HFSRT *P* = .48	5.3	0.5	-	FFRT vs. HFSRT *P* = .38	6.4	[2.4, 13.4]	-	FFRT vs. HFSRT *P* = .81
HFSRT^i^	9.4	1.6	-	HFSRT vs. SRS *P* = .24	5.2	1.4	-	HFSRT vs. SRS *P* = .72	7.7	[4.5, 12.2]	-	HFSRT vs. SRS *P* = .87
SRS^i^	10.3	2.2	-	SRS vs. FFRT *P* = .15	5.2	2.1	-	SRS vs. FFRT *P* = .73	6.2	[2.4, 10.0]	-	SRS vs. FFRT *P* = .23
Cumulative^ii^	11.2	2.1	.057	-	5.6	1.0	.55	-	2.2	[1.1, 4.0]	< .001 [1.9, 8.2]	-
FFRT^ii^	11.0	2.4	.095	FFRT vs. HFSRT *P* = .82	5.4	0.8	.38	FFRT vs. HFSRT *P* = .32	2.3	[0.9, 4.7]	.088 [0.78, 10.3]	FFRT vs. HFSRT *P* = .35
HFSRT^ii^	11.3	1.6	.045	HFSRT vs. SRS *P* = .34	6.4	0.9	.40	HFSRT vs. SRS *P* = .31	0.0	[0, 4.5]	.0076 [1.6, inf]	HFSRT vs. SRS *P* = .10
SRS^ii^	12.2	1.8	.11	SRS vs. FFRT *P* = .32	5.3	0.9	.92	SRS vs. FFRT *P* = .91	4.2	[0.9, 11.7]	.35 [0.6, 10.2]	SRS vs. FFRT *P* = .41

*SE*, standard error; *OS*, overall survival after re-irradiation; *PFS*, progression-free survival after re-irradiation; *RN*, radiation necrosis; *FFRT*, fully fractionated radiotherapy; *HFSRT*, hypofractionated stereotactic radiotherapy; *SRS*, stereotactic radiosurgery; *CI*, confidence interval; “cumulative” denotes combined statistic for all fractionation groups within a treatment group; *mo*, months

*Pairwise comparison between treatment groups

**Pairwise comparison within a treatment group

^i^Re-irradiation only group

^ii^Re-irradiation with bevacizumab group

Among patients receiving FFRT, the reRT treatment group had a mean OS of 8.7 ± 1.6 months, with PFS of 5.3 ± 0.5 months, and RN rate of 6.4% (95% CI [2.4%, 13.4%]). In comparison, the reRT + BVZ group had a mean OS of 11.0 ± 2.4 months (*P* = .095), with PFS of 5.4 ± 0.8 months (*P* = .38), and RN rate of 2.3% (95% CI [0.9%, 4.7%], *P* = .088) (Table 5).

Among patients receiving HFSRT, the reRT group had a mean OS of 9.4 ± 1.6 months, with PFS of 5.2 ± 1.4 months, and RN rate of 7.7% (95% CI [4.5%, 12.2%]). In comparison, the reRT + BVZ treatment group had a mean OS of 11.3 ± 1.6 months (*P* = .045), with PFS of 6.4 ± 0.9 months (*P* = .40), and RN rate of 0% (95% CI [0%, 4.5%], *P* = .0076) (Table 5).

Among patients receiving SRS, the reRT treatment group had a mean OS of 10.3 ± 2.2 months, with PFS of 5.2 ± 2.1 months, and RN of 6.2% (95% CI [2.4%, 10.0%]). The reRT + BVZ group had a mean OS of 12.2 ± 1.8 months (*P* = .11), mean PFS of 5.3 ± 0.9 months (*P* = .92), and RN rate of 4.2% (95% CI [0.9%, 11.7%], *P* = .097) (Table 5).

Tables 6 and 7 present the results of multivariate regression analysis assessing the significance of nine explanatory variables-of-interest accounting for the variance in OS and PFS, respectively. BVZ treatment status was the only significant predictor of OS (*P* = .041) (Table 6). No significant predictor variables were identified for PFS (Table 7).

**Table 6:** Multivariate linear regression analysis for overall survival after re-irradiation

Explanatory variable	Beta value	95% CI	*P* value
Age	0.23	[− 0.11, 0.57]	.16
GBM	0.43	[− 3.76, 4.63]	.83
HFSRT	− 1.22	[− 4.64, 2.20]	.46
FFRT	0.24	[− 4.77, 5.25]	.92
Bevacizumab	2.51	[0.11, 4.92]	.041
FU	0.0066	[− 0.24, 0.25]	.95
PTV	− 0.023	[− 0.066, 0.018]	.24
EQD2 RT	0.41	[− 0.62, 1.43]	.41
EQD2 reRT	0.0186	[− 0.039, 0.076]	.50

*GBM*, glioblastoma; *HFSRT*, hypofractionated stereotactic radiotherapy; *FFRT*, fully fractionated radiotherapy; *FU*, latency between irradiations; *PTV*, planned tumor volume; *RT*, initial radiotherapy; *reRT*, re-irradiation therapy; *EQD2*, equivalent dose in 2Gy per fraction; *CI*, confidence interval

**Table 7:** Multivariate linear regression analysis for progression-free survival after re-irradiation

Explanatory variable	Beta value	95% CI	*P* value
Age	0.11	[− 0.089, 0.30]	.23
GBM	1.36	[− 1.21, 3.94]	.24
HFSRT	1.17	[− 1.41, 3.75]	.31
FFRT	0.71	[− 3.23, 4.67]	.67
Bevacizumab	1.40	[− 0.36, 3.18]	.099
FU	0.020	[− 0.18, 0.21]	.81
PTV	− 0.00012	[− 0.036, 0.036]	.99
EQD2 RT	0.080	[− 0.59, 0.75]	.78
EQD2 reRT	0.015	[− 0.032, 0.061]	.47

*GBM*, glioblastoma; *HFSRT*, hypofractionated stereotactic radiotherapy; *FFRT*, fully fractionated radiotherapy; *FU*, latency between irradiations; *PTV*, planned tumor volume; *RT*, initial radiotherapy; *reRT*, re-irradiation therapy; *EQD2*, equivalent dose in 2Gy per fraction; *CI*, confidence interval

### Heterogeneity analysis

Analysis of study heterogeneity was performed for RN and OS (Supplementary Table 1, 2). Fixed and random effect model values are reported side by side. For both outcomes, studies were separated according to re-treatment protocols. For RN outcomes, heterogeneity was significant among FFRT (*Q*(2) = 10.82; *P* = .0045), HFSRT (*Q*(8) = 34.8; *P* < .0001) and SRS treatment groups (*Q*(12) = 54.66; *P* < .0001). For OS outcomes, heterogeneity was significant among FFRT + BVZ (*Q*(2) = 6.27; *P* = 0.044), HFSRT (*Q*(3) = 9.85; *P* = .02), and SRS (*Q*(8) = 45.73; *P* < .0001) groups.

### Risk of bias analysis

We assessed individual studies for risk of bias (RoB) using ten factors of study quality for reRT + BVZ studies (Supplementary Table 3), and nine parameters of study quality for reRT-only studies (Supplementary Table 4; see Methods for further details). The average RoB score for reRT + BVZ studies was 7.75 ± 1.83 (range: 4–10), and 7.31 ± 1.61 (range: 4–10) for reRT-only studies. We evaluated the effects of RoB scores on outcome measures using Pearson correlations for PFS and RN and meta-regression for RN (Supplementary Table 5). The only significant association of outcomes with RoB score was observed for reported PFS among the FFRT group (*r*(1) = − 1.0; *P* < .0001).

## Discussion

High-grade gliomas remain incurable with universal recurrence. Treatment of recurrent HGG is limited and heterogeneous in terms of treatment protocols and reported efficacy. Although salvage combination therapy with re-irradiation and adjuvant bevacizumab (BVZ) has mixed outcomes, multiple studies suggest it improved overall survival, progression-free survival, radiation necrosis, and tolerable toxicity in patients with recurrent HGG. The current study attempts to consolidate knowledge from the available published data on this topic.

The reported outcome data compares recurrent HGG patients receiving reRT with or without concomitant BVZ. We set strict inclusion criteria that eliminate any cohorts wherein more than 17.5% of patients received additional systemic therapies or adjuvant surgery at the time of reRT. We found that patients receiving BVZ had marginally improved average overall survival (*P* = .057) and significantly lower rates of radiation necrosis (*P* < 0.001) compared to patients receiving reRT alone. Stratifying by the reRT fractionation regimen, BVZ significantly improves OS (*P* = .045) and RN (*P* = .0076) for HFSRT patients. Despite similar trends towards improved OS and RN in reRT + BVZ patients undergoing FFRT and SRS, no effects reached statistical significance. In a multivariate analysis adjusting for median patient age, WHO grade (III vs. IV), reRT modality, latency between initial RT and reRT, planned tumor volume for radiation planning, EQD2 at reRT, and total EQD2 across initial RT and reRT, treatment with BVZ was the only significant predictor of improved overall survival and accounted for improved OS of 2.5 months on average (95% CI [0.1, 4.9], *P* = .041).

The foremost accepted therapeutic mechanism for BVZ is the mitigation of radiation necrosis secondary to irradiation-induced vascular dysfunction [81]. First, irradiation of glioma tissue causes vascular damage and subsequent hypoxia of the surrounding tissue. Subsequent upregulation of HIF-1α augments a milieu of pro-survival factors, including VEGF. VEGF overexpression yields aberrant neovascularization, which is highly permeable, resulting in perilesional edema and, ultimately, radiation necrosis [81]. Importantly, astrocytic glioma lineages have pronounced VEGF-mediated pathologic sequelae [47]. Furthermore, hierarchical grading of glioma tumors is strongly associated with VEGF expression predominating in GBM tumors [11].

Preclinical evidence describes a significant reduction in tumor volume and vascularization following RT with BVZ, which likely explains the reduced rates of RN in novel HGG patients [5, 27, 28, 39, 40]. BVZ exhibits radio-sensitizing effects by selectively targeting glial stem cells, which are otherwise minimally neutralized by radiation [4, 5]. BVZ thereby limits aberrant revascularization, a key mechanism in restricting further tumor growth [4, 25, 39]. By reducing VEGF expression, BVZ helps establish a normoxic niche, enhancing the cytotoxicity of radiation therapy [5, 28]. Thus, concomitant BVZ and irradiation have an advantageous synergistic therapeutic effect compared to BVZ administration alone [28, 39].

Clinically, there have been conflicting reports on the therapeutic efficacy of adjuvant BVZ in recurrent HGG patients. Our systematic review found that reRT + BVZ improves both RN and OS when compared to reRT alone. In a recent study, Fleischmann et al. [23] demonstrated that BVZ was significantly associated with decreased radiation necrosis and edema rates. Consistent with our data, Fleischmann et al. reported significant reductions in RN using BVZ in both univariate and multivariate analyses. Additionally, several other publications corroborate a moderate side effect profile for BVZ, with low or acceptable reported toxicity rates. Among studies included in our review, the reRT-only and reRT + BVZ treatment group had comparable toxicity rates of 21% and 25%, respectively.

Superior survival and functional outcomes are more commonly reported for women than men with HGG [73]. Stratified by re-radiation modality, gender distributions were comparable for FFRT (35% female) and HFSRT (28% female), reflecting known demographics for HGG patient populations in general [73]. However, the gender demographics among patients receiving SRS differed significantly between reRT and reRT + BVZ treatment groups (*P* = .002, Table 3). Despite a higher proportion of male patients, the median KPS score among our pooled reRT + BVZ SRS cohort was significantly higher than the reRT SRS cohort. Such inconsistency indicates a predilection for better-performing males in the reRT + BVZ group, limiting the interpretation of outcome differences for our pooled SRS cohorts.

Our reRT + BVZ groups had a higher proportion of grade III gliomas. While grade III gliomas are associated with favorable clinical outcomes compared to GBM [50], it is unclear whether BVZ portends a particular benefit for patients with grade III gliomas relative to patients with GBM. However, interpretation of BVZ’s role in outcomes for patients with grade III gliomas is limited, considering the updated WHO glioma classification [41]. In fact, IDH wild-type anaplastic astrocytomas have been shown to have similar clinical and molecular behavior as IDH wild-type GBM [9, 60]. Still, IDH wild-type AA constitutes the minority of grade III AA [60], and tumor grade was not associated with OS, PFS, or RN rate in our comparative univariate analyses. Similarly, tumor grade was not significantly associated with survival outcomes in our multivariate analysis. Most important, we found a significant beneficial effect of BVZ treatment on overall survival after adjusting for tumor grade in a multivariate linear regression analysis.

Balancing planned radiation treatment volumes with appropriate radiation doses is paramount to achieving safe and effective treatment. Higher doses destroy tumors more efficiently, albeit with a higher risk of side effects, particularly radiation necrosis [38]. In our pooled cohorts, there were no differences in total reRT dose between reRT- and reRT + BVZ-treated patients, whether they received SRS, HFSRT, or FFRT. Improved outcomes may be attainable with more aggressive radiation dosages in the setting of BVZ, given BVZ’s radioprotective effects [23, 27, 40]. To this point, both Clarke et al. [14] and Schernberg et al. [61] discuss the possibility of using BVZ to permit higher radiation doses with SRS and FFRT without undue radiation toxicity. Clarke et al. demonstrated an acceptable toxicity profile of a more aggressive SRS regimen (33 Gy in 3 fractions, EQD2 = 107 Gy) compared to a lower dose regimen (30 Gy in 5 fractions, EQD2 = 60 Gy). Similarly, Schernberg et al. reported improved survival associated with a more aggressive FFRT regimen for reRT (EQD2 > 50 Gy) compared to less aggressive dosing. Finally, Yasuda et al. [79] reported no radiation necrosis in patients with a more aggressive HFSRT radiation regimen of 42 Gy in 7 fractions (EQD2 = 84 Gy) in combination with BVZ. Notably, these authors described acceptable toxicity profiles despite exceeding cumulative doses over 100 Gy, a range traditionally associated with increased risk of radiation necrosis in the absence of BVZ [43, 67]. While we did not find a significant effect of the total dose (i.e., EQD2) at re-irradiation on patient outcomes, more rigorous studies are required to investigate the optimal volume range and radiation dose for each fractionation regimen supplemented with BVZ.

### Limitations

The retrospective nature of the majority of available studies limited our systematic review [70]. Additionally, the paucity of literature for reRT + BVZ treatment involving HFSRT and SRS limited our assessment of BVZ and reRT modality on clinical outcomes. Several papers reported combined data for HGG tumors and did not delineate between grade III and IV gliomas. Moreover, the heterogeneity in data reporting and treatment protocols prevented us from disaggregating important demographic, treatment, and clinical outcome variables in some cases. Partial or inconsistent reporting of BVZ-induced toxicities also prevented us from performing any rigorous synthesis of adverse outcomes from BVZ in this analysis.

Overall, study heterogeneity and risk of bias were thus major limitations of our approach and are inherent in any systematic review or meta-analysis. We attempted to address both these issues using robust meta-analytic modeling techniques (Supplementary Tables 1-5). Our RoB analyses demonstrated that obvious sources of study bias did not systematically skew reported study outcomes. However, we found that study heterogeneity was present within multiple treatment groups for both RN and OS outcomes. Comparing our simple pooling of median OS and RN rates using weighted means (Table 5) with the more rigorous meta-analytic quantification of pooled estimates incorporating random study effects (Supplementary Tables 1-2) in general showed good agreement in the pooled survival and RN estimates. The only major exception was OS in the FFRT group (8.7 months using weighted means vs. 14.3 months using a random-effects model). This discrepancy is explained simply by the fact that many studies did not report sufficient data to be amenable to the meta-analysis of median survival times (see Methods); thus, only a subset of reviewed studies contributed to the data reported in Supplementary Table 2. Still, our findings provide the most comprehensive synthesis for recurrent glioma outcomes after reRT - given the limitations in data reported in the existing literature - and suggest a possible benefit of BVZ for both OS and RN. However, improved estimation of clinical outcomes will require improved data reporting from clinical studies, which would ideally involve comprehensive datasets reported to shared electronic databases. Continued innovation in meta-analytic techniques may also push the limits of what can be achieved with the existing literature. For example, direct graphical estimation of individual data points from published survival curves might be used to glean more granular data from individual studies even when not reported directly by authors in the text. While not attempted here, such an approach may become more feasible in future work utilizing novel analysis software being pioneered for this purpose [52, 58].

Regarding radiation necrosis, accurate evaluation of anti-VEGF therapy response is also limited by imaging techniques, as conventional MRI does not reliably differentiate between pseudoprogression, tumor progression, and RN [78]. However, dynamic contrast-enhanced (DCE) perfusion MRI and diffusion-weighted imaging (DWI) are better able to differentiate between post-treatment radiation effect and actual tumor progression [21, 32, 49, 55]. Decreased tumor permeability and perfusion detected on DCE-MRI can reliably correlate with improved clinical outcomes after BVZ treatment [8, 29]. Such imaging results suggest that BVZ’s effects are consistent with the proposed vascular modulating theory. Recent reviews have further justified the more consistent use of perfusion MRI technology for evaluating HGG patients [49, 55, 61]. The lack of uniform objective characterization of tumor progression limits reliable determination of PFS and the potential benefits of novel therapeutic agents in slowing disease progression [12]. These limitations may have contributed to the lack of a significant benefit of BVZ on PFS in our multivariate analysis. As DCE and DWI technology improves, guidelines should be updated to standardize radiologic determination of tumor progression and RN.

Finally, most publications included in this study predated the revised 2016 WHO classification of CNS tumors; thus, IDH classification was not consistently reported. While this limits our interpretation of outcomes according to current standards, our study’s comparative nature does elucidate general correlations according to a classically utilized grading schema. Future studies should consistently report and compare the effect of BVZ on recurrent gliomas characterized by the updated 2016 WHO schema for gliomas.

## Conclusion

To our knowledge, this study is the most extensive and targeted systematic review evaluating the impact of bevacizumab on clinical outcomes in the setting of recurrent HGG treated with reRT. Our results suggest that reRT + BVZ may be associated with improved OS and lower RN rates than reRT alone. Upon multivariate analysis, treatment with BVZ was the only clinical variable significantly associated with improved OS. reRT + BVZ treatment had the most pronounced benefits in patients receiving HFSRT. Our findings suggest a potential benefit for reRT + BVZ, yet are limited by inconsistent data reporting and heterogeneity in study methodologies, the latter of which was also reflected in the heterogeneity of OS and RN outcomes reported across studies describing the same patient subgroups. Our findings support further randomized prospective studies to robustly assess the potential benefit of BVZ in patients with glioma and highlight the need for improved outcome reporting of published studies in this area. Future studies should clarify optimal reRT protocols and BVZ regimens (i.e., timing, dosing, and treatment duration). Finally, further work is needed to more accurately diagnose radiologic progression and RN in patients with recurrent glioma undergoing reRT.

## Supplementary information

ESM 1 (DOCX 36 kb)
