# Supplementary material for: Radiotherapy versus combination radiotherapy-bevacizumab for the treatment of recurrent high-grade glioma: a systematic review
Source: Acta Neurochir (Wien). 2021 Apr 2;163(7):1921–34. doi: 10.1007/s00701-021-04794-3 (PMC8195900; doi:10.1007/s00701-021-04794-3)
Supplement: Supplementary file 1 — (DOCX 36 kb) [file 701_2021_4794_MOESM1_ESM.docx]

| Treatment group | Radiation necrosis (%) | Heterogeneity Quantification | Test for Heterogeneity |
| --- | --- | --- | --- |
| FFRT | Fixed effects model:  6.4% [2.9%, 13.5%]  Random effects model:  5.2% [0.6%, 33.3%] | $\tau^{2}=2.31$  $H=1.84$  $I^{2}= 70.5\%$ | $Q\left( 2 \right)=10.82$  $P=.0045$ |
| FFRT+BVZ | Fixed effects model:  2.3% [1.1%, 4.7%]  Random effects model:  1.0% [0.1%, 8.9%] | $\tau^{2}=1.37$  $H=1.50$  $I^{2}= 55.8\%$ | $Q\left( 5 \right)=10.91$  $P=.0532$ |
| HFSRT | Fixed effects model:  7.7% [4.7%, 12.2%]  Random effects model:  3.9% [0.8%, 16.9%] | $\tau^{2}=3.26$  $H=3.33$  $I^{2}= 81.7\%$ | $Q\left( 8 \right)=34.8$  $P<.0001$ |
| HFSRT+BVZ | Fixed effects model:  1.8% [0.4%, 8.5%]  Random effects model:  1.8% [0.4%, 8.5%] | $\tau^{2}=0$  $H=1$  $I^{2}= 0\%$ | $Q\left( 2 \right)=0.01$  $P=.99$ |
| SRS | Fixed effects model:  8.0% [6.1%, 10.4%]  Random effects model:  4.4% [1.8%, 10.2%] | $\tau^{2}=1.39$  $H=2.26$  $I^{2}= 80.4\%$ | $Q\left( 12 \right)=54.66$  $P<.0001$ |
| SRS+BVZ | Fixed effects model:  4.2% [1.4%, 12.1%]  Random effects model:  4.2% [1.4%, 12.1%] | $\tau^{2}=0$  $H=1$  $I^{2}= 0\%$ | $Q\left( 2 \right)=1.51$  $P=.47$ |

**Supplementary Table 1.** Radiation necrosis results and study heterogeneity assessed using fixed and random effects meta-analysis in each treatment group

FFRT, fully fractionated radiotherapy; HFSRT, hypofractionated stereotactic radiotherapy; SRS, stereotactic radiosurgery; BVZ, bevacizumab;

**Supplementary Table 2.** Median overall survival results and study heterogeneity assessed using random effects meta-analysis with quantile estimation

| Treatment group | Median OS* (months) | Heterogeneity Quantification | Test for Heterogeneity |
| --- | --- | --- | --- |
| FFRT | Random effects model:  $14.3 \left[ 5.4, 23.2 \right]$ | Insufficient data | Insufficient data |
| FFRT+BVZ | Random effects model:  $11.9 \left[ 8.6, 15.4 \right]$ | $\tau^{2}=6.04$  $H^{2}= 3.16$  $I^{2}= 68.4\%$ | $Q\left( 2 \right)=6.27$  $P=.044$ |
| HFSRT | Random effects model:  $9.3 \left[ 6.8, 11.8 \right]$ | $\tau^{2}=3.63$  $H^{2}= 2.88$  $I^{2}= 65.3\%$ | $Q\left( 3 \right)=9.85$  $P=.02$ |
| HFSRT+BVZ | Random effects model:  $11.0 \left[ 7.3, 14.7 \right]$ | Insufficient data | Insufficient data |
| SRS | Random effects model:  $10.1 \left[ 8.5, 11.7 \right]$ | $\tau^{2}=3.48$  $H^{2}= 3.45$  $I^{2}= 71.0\%$ | $Q\left( 8 \right)=45.73$  $P<.0001$ |
| SRS+BVZ | Random effects model:  $14.4 \left[ 9.6, 19.2 \right]$ | Insufficient data | Insufficient data |

OS, overall survival (months);FFRT, fully fractionated radiotherapy; HFSRT, hypofractionated stereotactic radiotherapy; SRS, stereotactic radiosurgery; BVZ, bevacizumab;

*Represents median OS computed via quantile estimation accounting for random study effects across the subset of reviewed studies reporting minimum, median, and maximum patient survival times from reRT.

**Supplementary Table 3.** Risk of bias assessment of re-irradiation with bevacizumab studies included in this systematic review

| Study | | RoB Score | Study Method  (0,1) | Randomization  (0,1) | Blinding  (0,1) | Primary Chemotherapy (0,1,2) | | | Tumor Subtype (0,1) | | reRT Protocol (0,1,2) | Additional Immune Therapy w/ reRT  (0,1) | BVZ Regimen (0,1,2) | Follow-up  (0,1, 2) | RN Diagnosis  (0, 1) |
| --- | --- | --- | --- | --- | --- | --- | --- | --- | --- | --- | --- | --- | --- | --- | --- |
| Fleischmann 2019 ^23^ | 7 | | 0 | 1 | 1 | | 1 | 1 | | 1 | | 0 | 1 | 1 | 1 |
| Palmer^γ^ 2018 ^51^ | | 7 | 1 | 1 | 1 | | 2 | 1 | | 0 | | 0 | 0 | 0 | 0 |
| Palmer^γ^ 2018 ^51^ | | 7 | 1 | 1 | 1 | | 2 | 1 | | 0 | | 0 | 0 | 0 | 0 |
| Schernberg 2017 ^61^ | | 9 | 1 | 1 | 1 | | 1 | 1 | | 2 | | 0 | 1 | 1 | 0 |
| Back 2015 ^3^ | | 9 | 0 | 1 | 1 | | 1 | 1 | | 2 | | 0 | 2 | 2 | 1 |
| Hundsberger 2013 ^34^ | | 7 | 1 | 1 | 1 | | 1 | 1 | | 0 | | 0 | 1 | 1 | 1 |
| Yasuda 2018 ^79^ | | 9 | 1 | 1 | 1 | | 2 | 1 | | 1 | | 0 | 1 | 1 | 1 |
| Minniti 2015 ^46^ | | 5 | 1 | 1 | 1 | | 0 | 1 | | 0 | | 1 | 0 | 0 | 0 |
| Gutin 2009 ^29^ | | 4 | 0 | 1 | 1 | | 1 | 1 | | 0 | | 0 | 0 | 0 | 0 |
| Clarke 2017 ^14^ | | 10 | 1 | 1 | 1 | | 2 | 1 | | 1 | | 1 | 1 | 1 | 1 |
| Cabrera 2013 ^8^ | | 9 | 1 | 1 | 1 | | 2 | 1 | | 2 | | 1 | 0 | 0 | 0 |
| Cuneo 2012 ^18^ | | 10 | 1 | 1 | 1 | | 2 | 1 | | 1 | | 1 | 1 | 1 | 1 |

RoB, risk-of-bias; reRT, re-irradiation therapy; BVZ, bevacizumab; RN, radiation necrosis; γ, same publication with separate cohorts;

**Supplementary Table 4.** Risk of bias assessment in re-irradiation alone studies included in this systematic review

| Study | RoB Score | Study Method  (0,1) | Randomization  (0,1) | Blinding  (0,1) | Primary Chemotherapy (0,1,2) | Tumor Subtype (0,1) | reRT Protocol (0,1,2) | Additional Immune Therapy w/ reRT  (0,1) | Follow-up  (0,1, 2) | RN Diagnosis  (0, 1) |
| --- | --- | --- | --- | --- | --- | --- | --- | --- | --- | --- |
| Fleischmann 2019 ^23^ | 6 | 0 | 1 | 1 | 1 | 0 | 1 | 1 | 1 | 0 |
| Hundsberger 2013 ^34^ | 8 | 1 | 1 | 1 | 1 | 1 | 1 | 0 | 1 | 1 |
| Combs 2005 ^15^ | 6 | 1 | 1 | 1 | 2 | 0 | 1 | 0 | 0 | 0 |
| Gigliotti 2018 ^26^ | 7 | 1 | 1 | 1 | 0 | 1 | 1 | 1 | 0 | 1 |
| Zemlin 2018 ^80^ | 10 | 1 | 1 | 1 | 1 | 1 | 2 | 1 | 1 | 1 |
| Holt 2016 ^31^ | 7 | 1 | 1 | 1 | 1 | 0 | 1 | 0 | 1 | 1 |
| Dincoglan 2015 ^20^ | 5 | 1 | 1 | 1 | 0 | 0 | 0 | 1 | 1 | 0 |
| Ciammella 2013 ^13^ | 4 | 1 | 1 | 1 | 0 | 0 | 0 | 0 | 0 | 1 |
| Vordermark 2005 ^76^ | 8 | 1 | 1 | 1 | 2 | 0 | 2 | 1 | 0 | 0 |
| Voynov 2002 ^77^ | 9 | 1 | 1 | 1 | 1 | 1 | 1 | 1 | 1 | 1 |
| Hudes 1999 ^33^ | 9 | 0 | 1 | 1 | 2 | 1 | 2 | 0 | 1 | 1 |
| Shepherd 1997 ^67^ | 6 | 0 | 1 | 1 | 0 | 1 | 2 | 0 | 1 | 0 |
| Bir 2015 ^6^ | 6 | 1 | 1 | 1 | 1 | 0 | 0 | 0 | 1 | 1 |
| Pinzi 2015 ^57^ | 9 | 1 | 1 | 1 | 2 | 1 | 2 | 1 | 0 | 0 |
| Martinez-Carrillo 2014 ^42^ | 7 | 1 | 1 | 1 | 2 | 1 | 0 | 1 | 0 | 0 |
| Khalil 2013 ^36^ | 6 | 1 | 1 | 1 | 2 | 0 | 0 | 0 | 1 | 0 |
| Cuneo 2012 ^18^ | 9 | 1 | 1 | 1 | 2 | 1 | 1 | 1 | 1 | 0 |
| Skeie 2012 ^69^ | 8 | 1 | 1 | 1 | 2 | 0 | 1 | 1 | 0 | 1 |
| Elliott 2011 ^22^ | 6 | 1 | 1 | 1 | 0 | 1 | 0 | 1 | 1 | 0 |
| Torok 2011 ^74^ | 9 | 1 | 1 | 1 | 2 | 0 | 2 | 0 | 1 | 1 |
| Biswas 2009 ^7^ | 9 | 1 | 1 | 1 | 2 | 0 | 2 | 1 | 0 | 1 |
| Kong 2008 ^37^ | 7 | 1 | 1 | 1 | 2 | 0 | 1 | 1 | 0 | 0 |
| Combs 2005 ^16^ | 5 | 1 | 1 | 1 | 2 | 0 | 0 | 0 | 0 | 0 |
| Hall 1995 ^30^ | 10 | 1 | 1 | 1 | 2 | 1 | 2 | 0 | 1 | 1 |
| Shrieve 1995 ^68^ | 8 | 1 | 1 | 1 | 2 | 1 | 1 | 0 | 1 | 0 |
| Chamberlain 1994 ^10^ | 6 | 0 | 1 | 1 | 1 | 1 | 0 | 1 | 1 | 0 |

RoB, risk-of-bias; reRT, re-irradiation therapy; BVZ, bevacizumab; RN, radiation necrosis;

**Supplementary Table 5**. Assessment for risk-of-bias in outcome variables among included studies

| Treatment group and survival metric | RoB vs Outcome Variable |
| --- | --- |
| FFRT – median OS | $r\left( 1 \right)=0.99; P=.094$ |
| FFRT – median PFS | $r\left( 1 \right)=-1.0; P<.0001$ |
| FFRT – RN (%) | $= 0.924 \left[ -1.49, 3.34 \right]; P = .45$ |
| FFRT+BVZ – median OS | $r\left( 4 \right)=-0.21; P=.70$ |
| FFRT+BVZ – median PFS | $r\left( 4 \right)=0.91; P=.27$ |
| FFRT+BVZ – RN (%) | $= -0.05 \left[ -0.942, 0.849 \right]; P = .92$ |
| HFSRT – median OS | $r\left( 7 \right)=-0.43; P=.25$ |
| HFSRT – median PFS | $r\left( 2 \right)=-0.64; P=.36$ |
| HFSRT – RN (%) | $\beta=0.08 \left[ -0.50, 0.67 \right]; P=.78$ |
| HFSRT+BVZ – median OS | $r\left( 4 \right)=-0.84; P=.36$ |
| HFSRT+BVZ – median PFS | Insufficient data |
| HFSRT+BVZ – RN (%) | $= -0.03 \left[ -0.78, 0.72 \right]; P = .94$ |
| SRS – median OS | $r\left( 11 \right)=-0.33; P=.27$ |
| SRS – median PFS | $r\left( 5 \right)=-0.47; P=.28$ |
| SRS – RN (%) | $\beta=0.09 \left[ -0.28, 0.47 \right]; P=.63$ |
| SRS+BVZ – median OS | $r\left( 1 \right)=-0.83; P=.38$ |
| SRS BVZ – median PFS | $r\left( 1 \right)=-0.82; P=.39$ |
| SRS+BVZ – RN (%) | $\beta=0.55 \left[ -2.49, 3.6 \right]; P=.72$ |

FFRT, fully fractionated radiotherapy; HFSRT, hypofractionated stereotactic radiotherapy; SRS, stereotactic radiosurgery; BVZ, bevacizumab; OS, overall-survival from re-irradiation; PFS, progression-free survival from re-irradiation; RN, radiation necrosis; RoB, risk-of-bias;
